# Impact of geriatric comorbidity and polypharmacy on cholinesterase inhibitors prescribing in dementia

**DOI:** 10.1186/1471-244X-11-190

**Published:** 2011-12-06

**Authors:** Falk Hoffmann, Hendrik van den Bussche, Birgitt Wiese, Gerhard Schön, Daniela Koller, Marion Eisele, Gerd Glaeske, Martin Scherer, Hanna Kaduszkiewicz

**Affiliations:** 1 University of Bremen, Centre for Social Policy Research, Division Health Economics, Health Policy and Outcomes Research, Bremen, Germany; 2 Institute of Primary Medical Care, University Medical Center Hamburg-Eppendorf, Germany; 3 Institute of Biometrics, Hannover Medical School, Germany; 4 Institute of Medical Biometry and Epidemiology, University Medical Center Hamburg-Eppendorf, Germany

## Abstract

**Background:**

Although most guidelines recommend the use of cholinesterase inhibitors (ChEIs) for mild to moderate Alzheimer's Disease, only a small proportion of affected patients receive these drugs. We aimed to study if geriatric comorbidity and polypharmacy influence the prescription of ChEIs in patients with dementia in Germany.

**Methods:**

We used claims data of 1,848 incident patients with dementia aged 65 years and older. Inclusion criteria were first outpatient diagnoses for dementia in at least three of four consecutive quarters (incidence year). Our dependent variable was the prescription of at least one ChEI in the incidence year. Main independent variables were polypharmacy (defined as the number of prescribed medications categorized into quartiles) and measures of geriatric comorbidity (levels of care dependency and 14 symptom complexes characterizing geriatric patients). Data were analyzed by multivariate logistic regression.

**Results:**

On average, patients were 78.7 years old (47.6% female) and received 9.7 different medications (interquartile range: 6-13). 44.4% were assigned to one of three care levels and virtually all patients (92.0%) had at least one symptom complex characterizing geriatric patients. 13.0% received at least one ChEI within the incidence year. Patients not assigned to the highest care level were more likely to receive a prescription (e.g., no level of care dependency vs. level 3: adjusted Odds Ratio [OR]: 5.35; 95% CI: 1.61-17.81). The chance decreased with increasing numbers of symptoms characterizing geriatric patients (e.g., 0 vs. 5+ geriatric complexes: OR: 4.23; 95% CI: 2.06-8.69). The overall number of prescribed medications had no influence on ChEI prescription and a significant effect of age could only be found in the univariate analysis. Living in a rural compared to an urban environment and contacts to neurologists or psychiatrists were associated with a significant increase in the likelihood of receiving ChEIs in the multivariate analysis.

**Conclusions:**

It seems that not age as such but the overall clinical condition of a patient including care dependency and geriatric comorbidities influences the process of decision making on prescription of ChEIs.

## Background

International and national guidelines recommend the use of cholinesterase inhibitors (ChEIs) for mild to moderate Alzheimer's disease [1-3]. Pharmacological treatment with ChEIs has shown improvements in cognition and activities of daily living [4]. However, the clinical relevance of these treatment effects continues to be questioned [5,6].

In Germany, the ChEIs donepezil and rivastigmine are available since 1997 and 1998, and galantamine since 2001. Although in some countries donepezil is also approved for use in severe Alzheimer's dementia, in Germany ChEIs are only approved for mild to moderate Alzheimer's disease. The prescription volumes of ChEIs increased more than fivefold from 8.6 million defined daily doses (DDD) in 2001 to 46.8 million DDD in 2009 [7,8]. An increase was especially found in older patients [9]. Despite this rise of prescription volumes, the proportion of dementia patients treated with ChEIs in Germany is still low. Based on claims data of 2004-2006 van den Bussche et al. found that 13% of patients with incident dementia received ChEIs within the first year after diagnosis and that less than half of those treated were prescribed an appropriate dose [10]. Also based on administrative claims in the Lombardy Region in Italy Franchi et al. found that among estimated incident cases, the percentage of newly treated patients with ChEIs dropped from 12% in 2004 to 8% in 2007 [11]. In a comparison of ChEI treatment prevalences in 2004 across nine European countries Pariente et al. found a range between 3% in the Netherlands to 20% in France [12]. They found 7% treated patients in Germany and 6% in Italy, respectively. The authors attributed these variations between countries to different health care and reimbursement policies.

Factors promoting prescription of ChEIs found in the literature are younger age [11,13], living in a rural area in Germany, a lower number of comorbid conditions [10], contact with a neuropsychiatrist and a diagnosis of Alzheimer's disease in comparison to unspecified diagnoses and other specific dementias [10,14]. Based on a knowledge test Pentzek et al. stated that most general practitioners are aware of the positive expectations surrounding anti-dementia drugs, which are supposed to improve cognition and activities of daily living and to slow down progression [15]. However, for many physicians these expectations are too optimistic and differ with their own experiences. Probable factors impeding ChEI prescriptions have not yet been studied systematically. They are not mutually exclusive and refer to difficulties with the diagnosis of the dementia syndrome and type, presence of significant concurrent morbidity, adverse drug reactions, and fatalistic acceptance of the condition [13]. They all are positively related with age. Multimorbidity, usually associated with polypharmacy, may be the key to understanding the low prescription prevalences of ChEIs in patients with dementia. In the recent dementia guideline of the German College of General Practitioners and Family Physicians, multimorbidity is even listed as a potential reason for not further investigating the suspicion of dementia [16]. This must have implications for diagnosis, disclosure and treatment. However, the role of geriatric comorbidity and polypharmacy in the prescription of ChEIs has not yet been studied systematically. Therefore the main question of this study is:

Do geriatric comorbidity and polypharmacy influence the prescription of ChEIs in patients with dementia in Germany?

## Methods

### Design and study population

We used claims data of a cohort of 1,848 patients with incident dementia, which is described in detail elsewhere [10,17-19]. In brief, these patients were drawn from the Gmünder ErsatzKasse (GEK), a statutory health insurance company which insured 1.7 million people located in all regions of Germany (2% of the German population). We included only patients with a first diagnosis of dementia in outpatient care between the first quarter of the year 2005 and the first quarter of 2006. All patients included had a period free from this diagnosis of at least 4 quarters before the first quarter with such a code. Quarters had to be chosen because they form the basic time period for coding diagnoses in outpatient care in Germany. Patients were included if the following criteria were fulfilled:

• age of at least 65 years,

• at least one ICD-10 code for dementia from the following list (F00.x, F01.x, F02.0, F02.3, F03, G30.x, G31.0, G31.1, G31.82, G31.9, and R54) in outpatient care in at least 3 of 4 consecutive quarters,

• continuous insurance period in the year before and after the first code was recorded.

The quarter in which one of the codes appears for the first time is called the 'incidence quarter'. This and the following 3 quarters are referred to as the 'incidence year'. For this study, we used claims data for the incidence year.

### Cholinesterase inhibitors and covariates

Our dependent variable was at least one prescription of any of the three cholinesterase inhibitors (donepezil, rivastigmine, galantamine) in the incidence year. Our main independent variables were polypharmacy and measures of geriatric comorbidity. The concurrent use of multiple drugs is often termed polypharmacy, but there is no accepted international definition of this concept [20-22]. However, such a definition might be difficult because it has to be applied to different age groups, index diseases or populations. Therefore, we categorized the number of prescribed medications in our cohort into quartiles and operationalized polypharmacy in each patient (0-25%, 26-50%, 51-75% and 76-100%).

We used two measures of geriatric comorbidity: (1) data of the long term care insurance and (2) symptom complexes characterizing geriatric patients. Services from the German long term care insurance are provided to those who require support in the activities of daily living including personal hygiene, eating, mobility and - separate from personal care - housekeeping. There are three levels of care dependency related to the estimated time required for assistance indicating moderate (level 1), severe (level 2) and severest care dependency (level 3) [23,24]. If care dependency changed within the incidence year we considered the highest level in our analyses. Symptom complexes characterizing geriatric patients were derived from a consensus of several national geriatric associations in Germany. For instance, these 15 complexes include amongst others incontinence, risk of falls and dizziness, pressure ulcer as well as severe visual disturbances and hearing loss. We did not consider cognitive deficits since all of our patients would fulfil this criterion. The corresponding ICD-10 codes are published in Borchelt et al. [25] (see Table 1). Outpatient diagnoses documented in the incidence year (at least one quarter with a corresponding ICD-code) were used to assign the respective number of symptom complexes to each patient - characterizing his or her geriatric comorbidity.

**Table 1:** List of the symptom complexes characterizing geriatric patients and their corresponding ICD-10 codes used in this study (according to Borchelt et al. [25])

Symptom complexes characterizing geriatric patients	ICD-10 codes
Immobility	M96.8, M62.3, M62.5
Falls risk and dizziness	R26, R29.81, R42, H81, H82
Incontinence	R32, N39.3, N39.4, R15
Pressure ulcer	L89, L97, I83.0, I83.2, L98.4
Malnutrition	R64, E41, E43, E44
Disorders of fluid and electrolyte balance	E86, E87, R60
Depression and anxiety	F32, F33, F30, F31, F40, F41
Pain	R52, R51, N23, R10, M54, K08.88, F62.80, H57.1, M79.6, M25.5, R07.0-R07.4, N64.4, H92.0, F45.4, M75.8, K14.6
Neuropathies	R20, G50-G59, G60-G64
Frailty	R54
Severe visual disturbances and hearing loss	H53, H54, H52.4, H25, H28, H90, H91
Medication problems	Y57.9, X49.9
High risk of complications	Z98, Z48, Z43, T79-T89, Z99.2, I48
Delayed convalescence	Z54

We included sex and age as further independent variables. A dichotomous regional variable indicating living in an urban or rural area was created based on municipalities. The procedure of constructing this variable is described elsewhere [18]. We further assessed the number of physician contacts with neurologists and psychiatrists in the incidence year.

### Statistical analysis

After a descriptive characterization of the study cohort, the proportion of patients with at least one prescription of ChEIs was estimated. To study the relation between the prescription of ChEIs and polypharmacy, measures of geriatric comorbidity as well as other covariates, we fitted logistic regression models. First, univariate analyses were performed to determine the association between preselected variables and prescribing (model 1). The following variables were included: age (65-74, 75-84, 85+ years); sex (male, female); area of residence (urban, rural); quartile of number of prescribed medications (4 categories); level of care dependency (4 categories); number of symptom complexes characterizing geriatric patients (4 categories: 0, 1-2, 3-4, 5 and more) and number of contacts to neurologists/psychiatrists in the incidence year (5 categories: 0, 1-2, 3-5, 6-9, 10 and more). Next, these variables were entered in a multivariate model (model 2). Crude and adjusted odds ratios (OR) with 95% confidence intervals (95% CI) were estimated.

We used SAS for Windows version 9.2 (SAS Institute Inc., Cary, NC) for all statistical analyses.

The study was conducted according to the principles expressed in the Declaration of Helsinki. We considered the STROBE statement and the criteria of a national good practice guideline [26,27]. According to the Good Practice of Secondary Data Analysis, a national guideline for the use of administrative databases, no approval of an ethical committee is required [27].

## Results

### Characteristics of the study cohort

Baseline characteristics of the 1,848 patients with incident dementia are shown in Table 2. Individuals in the cohort are on average 78.7 years of age, and 47.6% are female. Most of them live in an urban environment (72.0%). Patients received on average 9.7 different medications (interquartile range: 6-13), and 44.4% are assigned to one of the three care levels. The most common symptom complexes characterizing geriatric patients are severe visual disturbances and hearing loss (47.7%), pain (46.1%), high risk of complications (35.9%), depression and anxiety (32.9%) and incontinence (24.0%). Virtually all patients (92.0%) are classified as having at least one of these symptom complexes. Altogether, 44.6% saw a neurologist or psychiatrist at least once within the incidence year.

**Table 2:** Characteristics of patients with incident dementia (n = 1,848)

Baseline characteristics	
Mean age, in years (SD)	78.7 (7.4)
Age groups, in years	
65-74	30.6%
75-84	47.1%
85+	22.3%
Sex	
Male	52.4%
Female	47.6%
Area of residence*	
Urban	72.0%
Rural	28.0%
Number of prescribed medications	
Mean (SD)	9.7 (5.7)
Minimum (Q0, 0th percentile)	0
First quartile (Q1, 25th percentile)	6
Second quartile (Q2, median, 50th percentile)	9
Third quartile (Q3, 75th percentile)	13
Maximum (Q4, 100th percentile)	51
Level of care dependency	
None	55.6%
1	20.7%
2	18.5%
3	5.2%
Symptom complexes characterizing geriatric patients	
Severe visual disturbances and hearing loss	47.7%
Pain	46.1%
High risk of complications	35.9%
Depression and anxiety	32.9%
Incontinence	24.0%
Falls risk and dizziness	21.9%
Neuropathies	17.4%
Disorders of fluid and electrolyte balance	15.4%
Pressure ulcer	9.5%
Frailty	3.9%
Others (immobility, malnutrition, medication problems, delayed convalescence)	2.7%
Number of symptom complexes characterizing geriatric patients	
0	8.0%
1-2	44.8%
3-4	34.5%
5+	12.7%
Contacts to neurologists/psychiatrists	
0	55.4%
1-2	9.0%
3-5	9.8%
6-9	12.2%
10+	13.6%

* missing values for 2 patients for which classification into the urban or rural group was not possible

### Prescribing of cholinesterase inhibitors

The proportion of patients who received ChEIs was 13.0%. Concerning the first prescription of this drug class in the incidence year, the majority was prescribed by neurologists and psychiatrists (60.2%) as well as internists and general practitioners (32.4%). The most frequently used substance was donepezil (47.3%), followed by galantamine (30.7%) and rivastigmine (22.0%).

### Factors associated with prescribing

The proportions of patients with at least one prescription of ChEIs stratified by covariates are presented in Table 3. This table also shows results of the univariate and multivariate logistic regression analyses. Crude prevalences and unadjusted odds ratios demonstrate that younger patients were more likely to receive a prescription (65-74 vs. 85+ years: 18.9% vs. 6.6%; OR: 3.32; 95% CI: 2.13-5.18). When stratified for sex, living in a rural vs. urban environment and for the number of prescribed medications, we found no significant differences in prescribing patterns. Patients not assigned to the highest care level were more likely to receive a prescription (e.g., no level of dependency vs. level 3: 15.7% vs. 3.1%; OR: 5.83; 95% CI: 1.82-18.61). The prescription prevalence decreased with increasing numbers of geriatric symptoms complexes (e.g., 0 vs. 5+ complexes: 22.3% vs. 7.7%). The number of contacts to neurologists and psychiatrists also had a strong influence on prescribing a ChEI. Only 4.2% of patients with no contact to these specialists received a prescription, this proportion increased to 37.3% in patients with 6-9 contacts and then decreased (21.5% in those with 10+ contacts).

**Table 3:** Logistic regression of factors associated with at least one prescription of cholinesterase inhibitors in the incidence year and characteristics of ChEI users vs.non-users

Characteristics	ChEI users (n = 241)	Non-users (n = 1,607)	Proportion of ChEI user (n = 1,848)	Model 1^a)^ OR_crude _(95% CI)	Model 2^b)^ OR_adj _(95% CI)
Age groups, in years					
65-74	107 (44.4%)	459 (28.6%)	18.9%	3.32 (2.13-5.18)	1.41 (0.85-2.34)
75-84	107 (44.4%)	763 (47.5%)	12.3%	2.00 (1.29-3.10)	1.30 (0.81-2.11)
85+	27 (11.2%)	385 (24.0%)	6.6%	1	1
Sex					
Male	137 (56.8%)	832 (51.8%)	14.1%	1.23 (0.93-1.61)	0.89 (0.65-1.21)
Female	104 (43.2%)	775 (48.2%)	11.8%	1	1
Area of residence*					
Urban	168 (69.7%)	1161 (72.3%)	12.6%	1	1
Rural	73 (30.3%)	444 (27.7%)	14.1%	1.14 (0.85-1.53)	1.48 (1.06-2.06)
Number of prescribed medications					
Q1 (0-6)	90 (37.3%)	520 (32.4%)	14.8%	1.43 (0.97-2.10)	0.93 (0.58-1.49)
Q2 (7-9)	59 (24.5%)	352 (21.9%)	14.4%	1.38 (0.91-2.10)	1.03 (0.64-1.65)
Q3 (10-13)	48 (19.9%)	372 (23.1%)	11.4%	1.06 (0.69-1.64)	0.79 (0.49-1.28)
Q4 (14+)	44 (18.3%)	363 (22.6%)	10.8%	1	1
Level of care dependency					
None	161 (66.8%)	866 (53.9%)	15.7%	5.83 (1.82-18.61)	5.35 (1.61-17.81)
1	41 (17.0%)	341 (21.2%)	10.7%	3.77 (1.14-12.44)	4.32 (1.26-14.75)
2	36 (14.9%)	306 (19.0%)	10.5%	3.69 (1.11-12.24)	3.97 (1.16-13.60)
3	3 (1.2%)	94 (5.8%)	3.1%	1	1
Symptom complexes characterizing geriatric patients					
0	33 (13.7%)	115 (7.2%)	22.3%	3.46 (1.87-6.41)	4.23 (2.06-8.69)
1-2	120 (49.8%)	707 (44.0%)	14.5%	2.05 (1.22-3.44)	2.52 (1.41-4.50)
3-4	70 (29.0%)	568 (35.3%)	11.0%	1.49 (0.87-2.55)	1.59 (0.89-2.84)
5+	18 (7.5%)	217 (13.5%)	7.7%	1	1
Contacts to neurologists/psychiatrists					
0	43 (17.8%)	981 (61.0%)	4.2%	1	1
1-2	16 (6.6%)	151 (9.4%)	9.6%	2.42 (1.33-4.40)	2.53 (1.37-4.65)
3-5	44 (18.3%)	137 (8.5%)	24.3%	7.33 (4.64-11.57)	7.86 (4.88-12.66)
6-9	84 (34.9%)	141 (8.8%)	37.3%	13.59 (9.04-20.43)	14.05 (9.14-21.59)
10+	54 (22.4%)	197 (12.3%)	21.5%	6.25 (4.07-9.60)	7.66 (4.85-12.11)

^a) ^crude models

^b) ^multivariate model adjusted for all other variables

* missing values for 2 patients for which classification into the urban or rural group was not possible

Odds ratios from the multivariate logistic regression model predicting at least one ChEI prescription are very similar to those of the univariate analyses, with a few exceptions. The effect of age was no longer statistically significant when adjusting for all other covariates. Living in a rural compared to an urban environment was associated with a significant increase in the likelihood of receiving ChEIs (OR: 1.48; 95% CI: 1.06-2.06), whereas in the univariate model, no significant relationship had been observed. The association of measures of geriatric comorbidity (care levels and symptoms characterizing geriatric patients) as well as the strong influence of contacts to specialists on the chance of being prescribed a ChEI remained in the multivariate analysis.

## Discussion

### Findings, comparison with other studies and interpretation

In this study using administrative data of a cohort of 1,848 patients with incident dementia, we found that contacts to specialists and measures of geriatric comorbidity are strongly associated with being prescribed a ChEI. Older patients were less likely to receive a prescription only in the univariate analysis, no significant relationship was observed in the multivariate model. Living in a rural area had an influence on prescribing - only in the multivariate model. The number of medications as a measure of polypharmacy was not associated with prescribing ChEI.

The univariate findings concerning age are in line with the results of Franchi et al., [11] and Lucca et al. [13] who both performed univariate analyses. The disappearance of a significant effect of age on ChEI prescribing in the adjusted model suggests that age as such is not an important factor for the decision to prescribe. Instead the overall clinical condition of a patient seems to have an influence. We interpret the levels of care dependency and the number of symptom complexes characterizing geriatric patients as proxies for functional and cognitive impairment, and thus as proxies for frailty. Frailty is usually described as a condition in which a critical number of specific impairments comprising mobility, strength loss and weight loss occur simultaneously [28]. Regarding the finding that measures of geriatric comorbidity have a strong negative influence on ChEI prescription two extreme interpretations are possible. On the one hand, persons convinced of the benefits of ChEI might see a discrimination against the frail, dependent and ill. On the other hand, persons with less optimistic expectations regarding ChEI might see the cautious prescription of ChEI for these patients as a sign of sensible consideration of probable benefits and disadvantages. However, we still do not exactly know how physicians develop their perceptions of the benefits and drawbacks of antidementia drugs and how they put them into practice.

We examined polypharmacy because it may lead to adverse effects, drug-drug interactions, medication errors as well as poor compliance [21,22] and physicians might be cautious when prescribing ChEIs to vulnerable patients with polypharmacy. The number of distinct medications is often used as a comorbidity measure for predicting mortality or hospitalizations [29,30]. The overall number of medications was not associated with prescriptions of ChEI. This might underline the hypothesis that the number of already prescribed medications has a much smaller impact on the decision to prescribe ChEIs than the overall clinical condition of a patient.

Besides the measures of geriatric comorbidity discussed above we suggest that contacts to specialists have a strong influence on being prescribed a ChEI. This has also been shown in other German studies [10,14]. We assume that the processes of further investigating the suspicion of dementia (including a referral to a specialist) go hand in hand with the considerations whether to prescribe ChEI or not. Furthermore, in Germany, general practitioners often involve specialists in order to disburden their own prescription costs [31]. Accordingly, we found that 60% of first prescriptions of ChEIs in our cohort were prescribed by neurologists and psychiatrists.

Concerning urban-rural differences, we have recently shown that the provision of primary practice seems to be equally given in both areas but that rural patients are less likely to consult neurologists or psychiatrists [18]. In rural areas, the distance to such specialists can be far and the transportation difficult for patients and caregivers which might be one explanation for this result. We did not find urban-rural differences in the crude model. After adjusting for contacts to specialists in the multivariate analysis, the likelihood of at least one prescription of a ChEI in the incidence year was higher for patients in rural areas. This finding is in line with Bohlken et al., who showed that the prescribed doses of antidementia drugs per neurologist or psychiatrist are higher in rural compared to urban areas [32]. In summary, rural dementia patients less often see a neurologist or psychiatrist than urban patients, but those who do, have a greater chance to be prescribed a ChEI. These points underline the importance of studying regional differences in health services utilization.

### Strengths and limitations

Administrative data allow studying real-world utilization patterns of unselected populations including also oldest old, institutionalized, frail and cognitively impaired individuals, which represent a large majority of demented patients. Field studies on dementia have to deal especially with selection bias concerning these factors [33,34]. Furthermore, field studies are much more expensive and contain smaller and regional samples, whereas claims data, like ours, contain information on a nationwide population including urban and rural patients. However, field studies can apply several diagnostic tests, physical and psychological examinations performed by specially trained professionals and therefore enable the researchers to validate the diagnosis of dementia, which was not possible in our study. We were further unable to distinguish between different types of dementia, since about half of the cohort received ICD-codes for unspecified dementia [10]. However, it is clinical reality in outpatient care in Germany that treatment decisions in dementia often are being made without having established an exact etiological diagnosis. Furthermore, we do not have information on dementia severity, which is assumed to be a relevant factor for the decision to prescribe ChEIs or not. It has to be kept in mind that we studied filled prescriptions and there might be patient or caregiver factors that influence the decision (not) to fill a prescription that are not captured in the data. We can not guarantee the validity of our algorithm used to identify incident cases of dementia. Since a health care contact and diagnostic awareness are prerequisites for a diagnosis, especially patients with mild dementia are less likely to be identified in claims data [35]. This seems to be underlined by the fact that some patients already received prescriptions of antidementia drugs before their incidence year [10]. However, we choose at least four dementia-free quarters followed by three out of four consecutive quarters with codes indicating dementia to increase the validity of diagnoses by avoiding transitory or erroneous diagnoses. On the other hand, compared to a much broader definition of dementia cases this results in a smaller sample size. Furthermore, these inclusion criteria allow us to study treatment patterns in a more homogenous cohort of patients. Validity problems can also occur for the symptoms characterizing geriatric patients which might lead to an underestimation of these diagnoses. Updates of the corresponding complexes have been recently published (http://www.geriatrie-drg.de/dkger/main/gtmm-2010.html) but we used the most recent version available during the study period. Finally, there are several differences between health insurance funds, for example with respect to age, sex, socioeconomic position and morbidity [36,37]. These differences might have an impact on the utilization of health care resources. Thus extrapolations of analyses of single funds to the whole German population should be performed with caution. However, there seems to be no obvious reason for treatment differences in patients with dementia between different health insurance funds.

## Conclusions

We suggest that a lack of contacts to specialists and geriatric morbidity patterns reduce the chance for patients with incident dementia of being prescribed a ChEI. It seems that not age as such but the occurrence of care dependency and geriatric comorbidities influences prescriptions. Polypharmacy was not associated with prescriptions of ChEI. This might further underline that the clinical condition of a patient plays an important role in the process of decision making. Our findings give insight into the decision process whether or not to prescribe ChEI and point at the need for further investigations of decision making processes regarding medication, especially for a vulnerable group such as dementia patients.

## Competing interests

The authors declare that they have no competing interests.

## Authors' contributions

FH and HK conceptualized the study design and wrote the paper. FH and BW performed the statistical analyses. All authors interpreted the data, critically revised the manuscript, read and approved the final version.

## Pre-publication history

The pre-publication history for this paper can be accessed here:

http://www.biomedcentral.com/1471-244X/11/190/prepub
